# Neuroinflammation contributes to autophagy flux blockage in the neurons of rostral ventrolateral medulla in stress-induced hypertension rats

**DOI:** 10.1186/s12974-017-0942-2

**Published:** 2017-08-23

**Authors:** Dongshu Du, Li Hu, Jiaxiang Wu, Qin Wu, Wenjing Cheng, Yuhong Guo, Ruijuan Guan, Yahui Wang, Xingxin Chen, Xanxia Yan, Danian Zhu, Jijiang Wang, Shutian Zhang, Yanfang Guo, Chunmei Xia

**Affiliations:** 1 Laboratory of Neuropharmacology and Neurotoxicology, Shanghai Key Laboratory of Bio-Energy Crops, College of Life Science, Shanghai University, Shanghai, 200444 People’s Republic of China; 2 Department of Physiology and Pathophysiology, Shanghai Medical College, Fudan University, Shanghai, 200032 People’s Republic of China; 3 Obstetrics and Gynecology Hospital of Fudan University, Shanghai, 200011 People’s Republic of China; 4 School of Basic Medical Sciences, Fudan University, Shanghai, 200011 People’s Republic of China; 5 Department of Pediatrics, Pudong Gongli Hospital, Shanghai, 200135 People’s Republic of China

**Keywords:** Neuroinflammation, Pro-inflammatory cytokines, Microglia activation, Autophagic flux, Stress, Hypertension

## Abstract

**Background:**

Neuroinflammation plays hypertensive roles in the uninjured autonomic nuclei of the central nervous system, while its mechanisms remain unclear. The present study is to investigate the effect of neuroinflammation on autophagy in the neurons of the rostral ventrolateral medulla (RVLM), where sympathetic premotor neurons for the maintenance of vasomotor tone reside.

**Methods:**

Stress-induced hypertension **(**SIH) was induced by electric foot-shock stressors with noise interventions in rats. Systolic blood pressure (SBP) and the power density of the low frequency (LF) component of the SAP spectrum were measured to reflect sympathetic vasomotor activity. Microglia activation and pro-inflammatory cytokines (PICs (IL-1β, TNF-α)) expression in the RVLM were measured by immunoblotting and immunostaining. Autophagy and autophagic vacuoles (AVs) were examined by autophagic marker (LC3 and p62) expression and transmission electron microscopy (TEM) image, respectively. Autophagy flux was evaluated by RFP-GFP-tandem fluorescent LC3 (tf-LC3) vectors transfected into the RVLM. Tissue levels of glutamate, gamma aminobutyric acid (GABA), and plasma levels of norepinephrine (NE) were measured by using high-performance liquid chromatography (HPLC) with electrochemical detection. The effects of the cisterna magna infused minocycline, a microglia activation inhibitor, on the abovementioned parameters were analyzed.

**Results:**

SIH rats showed increased SBP, plasma NE accompanied by an increase in LF component of the SBP spectrum. Microglia activation and PICs expression was increased in SIH rats. TEM demonstrated that stress led to the accumulation of AVs in the RVLM of SIH rats. In addition to the Tf-LC3 assay, the concurrent increased level of LC3-II and p62 suggested the impairment of autophagic flux in SIH rats. To the contrary, minocycline facilitated autophagic flux and induced a hypotensive effect with attenuated microglia activation and decreased PICs in the RVLM of SIH rats. Furthermore, SIH rats showed higher levels of glutamate and lower level of GABA in the RVLM, while minocycline attenuated the decrease in GABA and the increase in glutamate of SIH rats.

**Conclusions:**

Collectively, we concluded that the neuroinflammation might impair autophagic flux and induced neural excitotoxicity in the RVLM neurons following SIH, which is involved in the development of SIH.

**Electronic supplementary material:**

The online version of this article (doi:10.1186/s12974-017-0942-2) contains supplementary material, which is available to authorized users.

## Background

Evidence shows that chronic psychosocial stresses play important roles in the development of hypertension, cardiovascular disease, and stroke [1]. Our previous study demonstrated that electric foot-shock stressors with noise interventions induced a significant enhancement of systolic blood pressure (SBP) in rats, which caused stress-induced hypertension (SIH) [2–4]. Activation of various neurogenic pathways such as the sympathetic nervous system (SNS) is pivotal in triggering stress-induced cardiac and vascular events [5]. Regulation of BP by the central nervous system depends on coordinated activities of highly interconnected neuronal networks [6, 7]. The rostral ventrolateral medulla (RVLM) is the main integration center for regulating sympathetic outflow which is involved in the neural mechanism of hypertension [8, 9].

Microglia are the principal neuroinflammatory cells in the central nervous system (CNS) parenchyma. Inflammatory events are initiated in the CNS upon sensing danger-associated molecular patterns (DAMPs) or pathogen-associated molecular patterns (PAMPs) by resident microglia and astrocytes [10]. In addition, these mediators can act in an autocrine/paracrine manner to activate neighboring microglia, astrocytes, neurons, or oligodendrocytes. The consequences of cytokine action vary between each cell type and can include perpetuating inflammatory mediator release, altering target cell function, or inducing cell death [11]. They are involved in several pathophysiological functions, including an unexpected impact on synaptic transmission and neuronal excitability. Hypertension induced by either angiotensin II (AngII) or l-N(G)- nitro-l-arginine methyl ester (L-NAME) is accompanied by microglial activation as manifested by microgliosis and pro-inflammatory cytokines (PICs) upregulation [12]. Emerging evidence supports PICs of tumor necrosis factor-α (TNF-α), nuclear factor kappa B (NF-κB) activation, and [12, 13] oxidative stress [14] are the important mediators in the brain involved in the neural mechanism of hypertension and cardiovascular disorders in both animal models and human diseases [15].

It was reported that redox-sensitive endoplasmic reticulum stress and activation of autophagy in the RVLM contribute to oxidative stress-associated neurogenic hypertension [16]. Abnormal autophagy has been linked to many pathophysiological events and is involved in disease development [17, 18]. Autophagosome formation and maturation are highly regulated by a series of distinct steps controlled by autophagy-related proteins, and ultimately, the autophagosome is degraded being fused with endocytic and lysosomal compartments [19]. The lysosome dysfunction is one of the reasons for autophagy dysfunction [20]. The term “autophagic flux” is often used to describe the overall process encompassing autophagosome assembly, substrate delivery, and degradation within the lysosome. The conversion from LC3 I to LC3 II is considered a good marker of autophagic activity as LC3 II is associated with autophagosomal membranes [21, 22]. Poly-ubiquitin binding protein sequestosome 1 (SQSTM1, p62) tends to accumulate when autophagy is inhibited while it tends to decrease when autophagy is induced [19, 20]^.^ Thus, monitoring levels of p62 in some settings is useful as a marker of autophagy flux [23].

Chronic inflammation, as seen in several lysosomal storage diseases, impairs macromolecule degradation following endosome-lysosome and phagosome-lysosome fusion and autophagy, ultimately disrupting cellular homeostasis [24]. It was reported that neuroinflammation and autophagy impairment led to neuronal dysfunction and associated deficits in hyperglycemia-induced neuronal injury in experimental models of diabetic neuropathy [25].

Our present study design is based on the following rationales. First, the RVLM is the main sympathetic output for heart and blood vessels playing important roles on autonomic control. Second, electric foot-shock stressor with noise interventions in rats is an established animal model of SIH with strong neurogenic components. Targeting of autophagy disorders associated with inflammatory cytokines, and related signaling molecules, might be considered a novel option for the development of therapies in SIH.

We observed that neuroinflammation impaired autophagic flux in SIH rats. Minocycline, an anti-inflammatory antibiotic, induced a hypotensive effect with attenuated microglia activation and decreased PICs in the RVLM of SIH rats, Furthermore, SIH rats had higher levels of glutamate and lower level of gamma aminobutyric acid (GABA) in the RVLM, while minocycline attenuated the decrease in GABA and the increase in glutamate of SIH rats.

Our results suggest that neuroinflammation might induce the blockage of autophagic flux and cause neural excitotoxicity in the RVLM neurons of rats following SIH. Autophagy flux blockage in the neurons of the RVLM might be involved in the pathogenesis and development of SIH.

## Methods

### Animal preparations

Seventy-two 8-week-old male Sprague-Dawley rats weighting 250–300 g were obtained from the animal center of Fudan University. They were housed at a 12 h light/dark cycle with free access to food and water; room temperature was maintained between 23 and 24 °C. All experimental procedures conformed to both Fudan University and international guidelines on the ethical use of animals; all efforts were made to minimize the number of animals used and their suffering. The rats were divided into three groups (*n* = 24): a normotensive rats group (control), SIH group, and SIH plus minocycline application (Mino + SIH) group. The SIH model was established as previously described [2–4]. Briefly, rats in the stressed groups were placed in a cage (22 cm × 22 cm × 28 cm) with a grid floor and subjected to electric foot shocks. The delivery of intermittent electric shocks (35–75 V, 0.5 ms in duration) through the grid floor every 2–30 s was randomly controlled by a computer. Noises (range, 88–98 dB) produced by a buzzer were given synchronously as a conditioned stimulus. The computer program was designed to give two types of stimuli: noises plus electric foot shocks or noises only, both of which were delivered randomly. On day 7 when SBP and heart rate (HR) of the stressed groups increased to a nearly stable level, the computer linked to the stressor apparatus was adjusted, gradually decreasing electric foot-shock times and prolonging the interval between shocks until only noise stimuli remained. The control group of rats underwent sham stress; they were put into the cage for the same period of time, but were neither subjected to foot shocks nor noises. Rats were subjected to stress for 2 h twice daily for l4 consecutive days. Two hours after being subjected to stress, SBP was consciously measured using the tail-cuffed method. Measurements were repeated three times and the average value was taken as the SBP (Additional file 1: Figure S1).

### Hemodynamic measurements

Before the rats were sacrificed, blood pressure was measured via a femoral artery cannula using a pressure transducer and a polygraph (Model SMUP-A, Department of Physiology and Pathophysiology, Shanghai Medical College of Fudan University, Shanghai, China). The HR was derived automatically from the arterial blood pressure phasic wave by the computer. During the experiment, body temperature was measured with a rectal thermometer and maintained at 37.5 ± 0.5 °C by a temperature controller (H-KWDY-III, Quanshui Experimental Instrument, Chengdu, China). Arterial blood PaO_2_ and PaCO_2_ were periodically monitored using a blood gas analyzer (EasyBloodGas, Medica Corporation, Bedford, MA, USA) and maintained within normal limits. Arterial blood pH was maintained between 7.35 and 7.45.

### Power spectral analysis of SAP signals

Power spectral analysis of SBP signals was performed in animals with sodium pentobarbital (50 mg kg^−1^, i.p.) anesthesia. SBP signal was monitored for 30 min by femoral artery cannulation. Power spectral analysis of SBP signals was performed by fast Fourier transform. The low frequency (LF, 0.25–0.8 Hz) component of the SBP spectrum was taken as it was originally from the RVLM [26, 27], and reflected the prevalence of sympathetic nerve activity to the vessels.

### Intracisternal implantation of osmotic minipump and agent infusion

The procedures were performed as similar study [15]. Briefly, the animals were anesthetized with pentobarbital sodium (50 mg kg^−1^, i.p.), then the osmotic minipump was implanted into the cisterna magna for infusion of minocycline. A midline dorsal neck incision was made, and the dura mater between the foramen magnum and C1 lamina was exposed following dissection of muscles. The dura was perforated with a 22-gauge steel needle. After observation of cerebrospinal fluid leakage from this hole, a PE-5 catheter (Clay Adams, Sparks, MD) was advanced for 5 mm into the cisterna magna. The catheter was sealed to the dura with tissue glue and incision was closed with layered sutures. The outer end of the catheter was connected to a micro-osmotic minipump (Alzet 1007D, DURECT Co., Cupertino, CA), which was placed under the skin in the neck region. The osmotic minipump (implanted SC) was connected to the infusion cannula via the catheter tube delivering agent. Inhibitor of microglial activation minocycline (Mino, 0.3 μl/h, Sigma-Aldrich, St. Louis, MO) was delivered by osmotic minipump for 1 week on the basis of published in vivo [15, 28, 29]. Animals received procaine penicillin (1000 IU, IM) injection postoperatively, and only animals that showed progressive weight gain after the operation were used in subsequent experiments. Control infusion of artificial cerebrospinal fluid (aCSF) was served as volume and vehicle control. Minocycline solution was prepared by dissolving 172 ng minocycline in 1 mL normal aCSF.

### Injections of adeno-associated virus-LC3 into the RVLM

In the process of autophagosome synthesis, the cytoplasmic localization of microtubule-associated protein 1 light chain 3-I (LC3-I) is conjugated with phosphatidylethanolamine and becomes the membrane-bound form LC3-II, which is located in both the outer and inner membranes of the autophagosomes. Due to this property, the most widely used assay for monitoring autophagosomes is the observation and quantification of fluorescent-tagged LC3 (such as GFP-LC3, RFP-LC3) puncta LC3-II by fluorescence microscopy [20]. Monitoring autophagic flux is important for the analysis of autophagy. Tandem fluorescent-tagged LC3 (tf-LC3) is a convenient assay for monitoring autophagic flux based on different pH stability of monomeric red fluorescence protein-enhanced green fluorescence protein-LC3 (mRFP-EGFP-LC3) proteins [20]^.^

The gene transfer of tf-LC3 and/or GFP-LC3 into the RVLM was performed by microinjection bilaterally into the nucleus of adeno-associated virus (AAV) RFP-GFP-tandem fluorescent LC3/GFP-LC3 14 days prior to stress application. An adenoviral suspension containing 5 × 10^8^ plaque-forming units (pfu)/100 nl mediated gene transferring into the RVLM was administered into each injection site over 10–15 min by pressure through a glass micropipette.

Intra-RVLM microinjection and hemodynamic measurements were performed as described previously [2–4]. Briefly, the rats were anesthetized with a mixture of urethane and chloralose comprised of 140 g urethane, 7 g chloralose, and 7 g borax per 1 l normal saline in an intraperitoneal dose of 7 ml/kg. Tracheas were intubated with polyethylene tubes, and the animals spontaneously breathed room air. Heads of the rats were affixed on a stereotaxic apparatus (Neurostar Tubingen, Germany) and flexed to an angle of approximately 45°. The occipital bone was carefully removed to expose the fourth ventricle, the floor of which was kept horizontally. A glass micropipette was inserted into the RVLM (1.5–1.9 mm ahead of the obex, 1.5–2.0 mm right of the midline, and 6.6–7.0 mm deep from the dorsal surface of the cerebellum) according to the atlas of Paxinos and Watson [30]. Following the microinjection, the muscle layers covering the fourth ventricle were sutured. Body temperature was maintained at 37 °C with heating pads until the animals had recovered from surgery. The rats were allowed to recover in their home cages with free access to food and water. Only animals that showed progressive weight gain after the gene transfer were used in subsequent experiments. Before the microinjection of virus into the RVLM, the sites were identified by monitoring BP after local injection of a small dose of L-glutamate.

At the end of the experiment, 2% Pontamine Blue Dye (100 nl) was microinjected to confirm injection site accuracy within the RVLM. The animals were scarified by an intraperitoneal overdose injection of composite anesthetic agents, and the brain was removed. After the rat brain was fixed in 10% formalin for 7 days, frozen brain cross-sections (30 μm) were made and stained with 1% Neutral Red to identify the microinjection sites as in our previous study [9]. The location of each study site was identified and mapped on diagrams of the rat brain (additional file 2: Figure S2 A and B) according to the atlas of Paxinos and Watson [30].

AAV9-mRFP-GFP-LC3/GFP-LC3 was purchased from Hanbio Co. Ltd. Shanghai China. In brief, the novel reporter fusion protein, tandem fluorescent-tagged LC3, mRFP-GFP-LC3 is delivered through an adeno-associated virus (AAV) (Hanbio, Shanghai, China). The GFP signals were attenuated in the lysosomal acidic conditions, whereas mRFP fluorescence was relatively stable after the fusion of autophagosomes with lysosomes. This is because the pKa value of GFP was relatively higher than mRFP (pKa^GFP^ = 6.0, pKa^mRFP^ = 4.5). Therefore, this tandem-tagged fluorescent protein showed merged GFP and mRFP fluorescence in the autophagosomes, but only exhibited mRFP fluorescence in lysosomes [31]. All injections were made in a volume of 3 μl within 1 min according to the manufacturer’s instructions. After 2 weeks infection, the animals were scarified and the brain was removed. The section of the RVLM was made to identify the fluorescent dying. Images were taken under a Fluorview FV300 laser scanning confocal microscope (Olympus, Tokyo, Japan). The yellow and red LC3 puncta were manually counted.

### Collection of tissue samples from the RVLM

At various time intervals after the experimental treatment, rats were sacrificed with an overdose of pentobarbital sodium and perfused intracardially with warm saline. The brain was rapidly removed and immediately frozen on dry ice. the Medulla oblongata covering the RVLM was blocked between 0.5 and 1.5 rostral to the obex, which was adopted from the atlas of Watson and Paxinos [30], and served as the anatomical landmark. These coordinates were selected to cover the extent of the ventrolateral medulla in which functionally identified sympathetic premotor neurons reside. Both sides of the ventrolateral medulla covering the RVLM (approximately at 1.5 to 2.5 mm lateral to the midline and medial to the spinal trigeminal tract) were collected by micropunches with a 1-mm inner diameter burr. There were 1–5 medullary tissues collected from the same experimental groups and the tissues were pooled and stored at −80 °C prior to mRNA or protein analysis.

### RNA extraction and quantitative real-time polymerase chain reaction

Total RNA from the RVLM was extracted using an RNA extraction kit RNAqueous (Ambion, TX) according to the manufacturer’s protocol. All RNA isolated was quantified by spectrophotometry and the optical density 260/280 nm ratio was determined. cDNA was synthesized using High Capacity cDNA Reverse Transcription Kit (Applied Biosystems, ABI). Following reverse transcription, quantitative real-time PCR was performed on StepOnePlus Real-Time PCR Systems (Applied Biosystems, ABI) under a condition of 40 cycles of 95 °C for 15 s and 60 °C for 1 min, using SYBR Green as indicator. The level of target mRNA was normalized against the expression of the internal control 18S in the same sample that was calculated with the DCt method. The primer pairs for amplification of total IL-1β cDNA (GenBank accession number NM_031512.2) were 5′-GTTACCGTTACTACTCCA T-3′ for the forward primer, and 5′-TGGAGTAGTAACGGTAAC-3′ for the reverse. TNF-α cDNA (GenBank accession number L00981.1) were 5′-GGCGAACCACCAAACG-3′for the forward primer, and 5′-CGTTTGGTGGTTCGCC-3′ for the reverse. β-actin cDNA (GenBank accession number V01217.1) were 5′-ATGGTGGGTATGGGTCAGA-3′ for the forward primer, and 5′-TACCACCCATACCCAGTCT-3′ for the reverse. The expression level of target mRNA in the control group from each independent experiment was considered as 1, and the relative expression level of target mRNA in experimental groups was adjusted as a ratio to its control in each independent experiment and expressed as fold changes.

### Western blot analysis

Total, cytosolic, or mitochondrial protein extract from the RVLM homogenates was used to analyze protein expression by Western blot. In brief, proteins (50 μg for total protein, 20 μg for cytosol, or 10 μg for mitochondria) from the RVLM were separated by using 10–12% SDS-PAGE and transferred to the PVDF membrane. The primary antiserum used for Western blot analysis included a mouse monoclonal antiserum against LC3B (1:1000; Invitrogen, Carlsbad, CA), (1:1000; Wako, Tokyo, Japan), a rabbit polyclonal antiserum against p62 (1:1000; Abcam, Cambridge, UK), TNF-α (1:1000; Calbiochem-Novabiochem), and Iba1 (1:1000; Abcam, Cambridge, UK). This was followed by incubation with horseradish peroxidase-conjugated goat anti-rabbit IgG or goat anti-mouse IgG (Jackson ImmunoReserach, West Grove, PA). Specific antibody-antigen complex was detected using an enhanced chemiluminescence Western blot detection system (NEN Life Science Products, Boston, MA). Cytosolic (LC3-I) and membrane-bound (LC3-II) LC3 proteins were identified based on its molecular size (LC3-I, 18 kDa; LC3-II, 16 kDa). The amount of detected protein was quantified by the Photo-Print Plus software (ETS Vilber-Lourmat, France), and was expressed as the ratio to β-actin protein.

### Double immunofluorescence staining and laser confocal microscopy

The procedures for double immunofluorescence staining were modified form our previous report [6]. In brief, free-floating 30-μm sections of the medulla oblongata containing the RVLM were incubated with a mouse monoclonal antiserum against LC3B (1:1000; Invitrogen, Carlsbad, CA), a rabbit polyclonal antiserum against p62 (1:1000; Abcam, Cambridge, UK), LAMP2 (1:400; Abcam, Cambridge, UK), NeuN (1:1000; Calbiochem-Novabiochem), and CD11b (1:1000; Cell Signaling, Danvers, MA), which is a specific marker for microglia for 24 h at 4 °C. The sections were subsequently incubated concurrently with a goat anti-rabbit IgG conjugated with Alexa Fluor 488, or a goat anti-mouse IgG conjugated with Alexa Fluor 568. No specific immunoreactivity was observed in control sections, which were incubated without a primary antibody. Viewed under a Fluorview FV300 laser scanning confocal microscope, immunoreactivity exhibited green fluorescence or manifested red fluorescence. The co-localization of red and green fluorescence on merged images indicated the presence of immunoreactivity in neurons or microglia. For quantification of immunoreactive cells in the RVLM, the medullary sections that cover the caudal-to-rostral axis of the RVLM were collected at 105-μm intervals. The immunoreactive cells on both sides of the RVLM from all collected sections were counted by two independent individuals in a single-blind fashion. Total number of counted cells was divided by the number of sections to represent the average number of immunoreactive cells on both sides of the RVLM per section.

### Microglia morphology analysis and quantification

Microglia were detected immunohistochemically by the presence of the marker protein CD11b (clone OX-42) which increases markedly upon activation of microglia [32]. Morphological analysis and quantification of microglia was performed with a light microscope using 400 times magnification. In each section, the total number of microglia was counted unilaterally in squares measuring 0.2 × 0.2 mm in size. In areas adjacent to the RVLM, a similar analysis was performed. The numbers of activated microglia counted were expressed as a percentage of the total number of microglia (activated + non-activated) counted in each region analyzed and averaged per section. Counting was performed by a single observer blinded to the treatment. Non-activated microglia were identified by their small soma from which there emanated extensive, highly branched, long, thin processes. Activated microglia were defined by three main criteria [32]: (1) stronger immunohistochemical staining for the marker CD11b, (2) the presence of a clearly enlarged soma, and (3) marked changes in the appearance of the processes which were now reduced in number, but considerably thicker and shorter giving a stubby appearance (Additional file 3: Figure S3).

### Transmission electron microscopy

To investigate the autophagy process, the RVLM were examined via transmission electron microscopy (TEM). The rats in different groups were deeply anesthetized and transcardially perfused with 0.1 M PBS, followed by 4% paraformaldehyde and 1% glutaraldehyde. The RVLM were cut into 1-mm transverse sections and maintained in the same fixative overnight. The sections were subsequently immersed in 1% osmium tetroxide (2 h), dehydrated in graded ethanol, and embedded in epoxy resin. Ultrathin sections (60–70 nm) were obtained using an ultramicrotome; these sections were then post-stained with uranyl acetate and lead citrate and examined using a TEM (Tenai G2 Spirit; Hillsboro, OR, USA).

### Measurement of the RVLM tissue levels of glutamate and GABA, as well as plasma NE

Tissue levels of glutamate and GABA and plasma levels of norepinephrine (NE) were measured using high-performance liquid chromatography (HPLC) with electrochemical detection as our previous publications [2] and the similar study described [33, 34].

#### Statistics

Data are expressed as means ± SEM. For experiments that involved two groups of samples, Student’s unpaired *t* test was used. For experiments that involved multiple groups, one-way or two-way analysis of variance with repeated measures were used to assess group means. This was followed by the Tukey’s multiple range tests for post hoc assessment of individual means. *P* < 0.05 was considered statistically significant.

## Results

### Stress-induced microglia activation in the RVLM of SIH

**Fig. 1:**
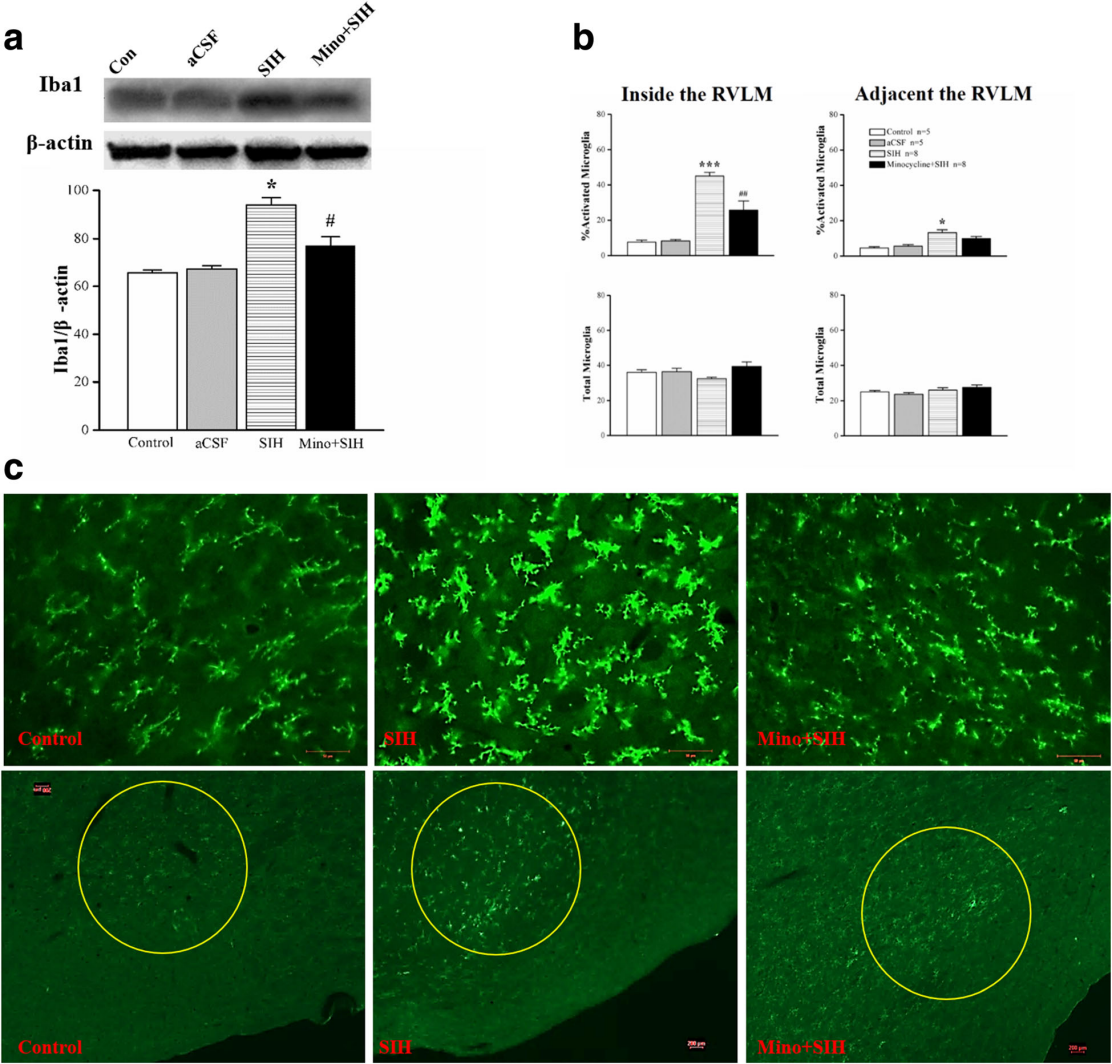
Representative gels (*inset*) or densitometric analysis (**a**) of protein level of ionized calcium-binding adaptor molecule-1 (Iba-1) detected from RVLM of SIH rats or age-matched normotensive rats or IC infusion of minocycline for 1 week in SIH rats. Microglial activation expressed as a percent of total microglia (**b**) in RVLM or adjacent to it and total number of microglia (activated + non-activated) in the same region. Photomicrographs showed microglia (**c**) taken from the RVLM of rats in different groups. The outline of the RVLM is highlighted by the *dashed lines*. Values are mean ± SEM of quadruplicate analyses on samples pooled from five to eight animals in each group. *P < 0.05 vs. control rats, and #*P* < 0.05 vs. the SIH group in the post hoc Scheffé multiple range analysis. *RVLM* rostral ventrolateral medulla. *Bar* = 50 μm in the top and 200 μm at the bottom panel of **c**

Ionized calcium-binding adaptor molecule-1 (Iba-1) is a calcium-binding protein specifically expressed in macrophage/microglia. Chronic stress increased Iba-1 protein expression, and treatment with minocycline into the cisterna magna attenuated this effect (Fig. 1a). In control rats, there were few microglia (approximately 10%) with an activated morphology in the RVLM. There was significant increase in the proportion of activated microglia in SIH rats compared to the control group. The proportion of activated microglia in the SIH averaged approximately 40–50% (Fig. 1b). Minocycline significantly attenuated the proportion of activated microglia by more than 50%. In the area adjacent to the RVLM, the average number of microglia counted was no significantly different between different groups (Fig. 1b). The morphology of microglia in different groups was shown in Fig. 1c.

### Suppression of activated microglia in the RVLM attenuated stress-induced PICs releasing in SIH rats

**Fig. 2:**
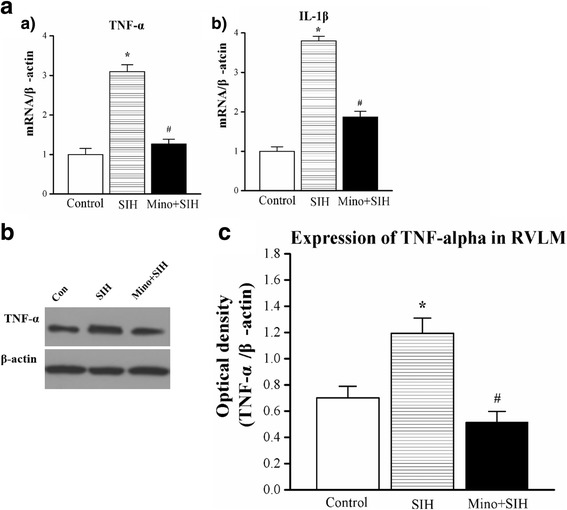
Effect of minocycline on interleukin (IL)-1β and tumor necrosis factor (TNF)-α expression in the RVLM. *Bar graphs* illustrate the expressions of IL-1β or TNF-α mRNAs (**a**) in the RVLM of control, SIH, minocycline only, and minocycline + SIH groups. Representative photomicrographs of Western blot (**b**) and densitometric analysis (**c**) for TNF-α in RVLM. Values are mean ± SEM of quadruplicate analyses on samples pooled from five to eight animals in each group. *P < 0.05 vs. control rats, and #*P* < 0.05 vs. the SIH group in the post hoc Scheffé multiple range analysis

Chronic stress significantly upregulated basal levels PICs expression in the RVLM. IL-1β and TNF-α (Fig. 2a) mRNA levels increased 3.8- and 3.0-fold in the RVLM of SIH, respectively. Western blot showed TNF-α protein was increased in SIH rats (Fig. 2b, c). Intracisternal infusion of minocycline could significantly attenuate the upregulation of PICs in the RVLM of SIH rats.

### Attenuation of microglial activation via intracisternal infusion of minocycline showed an antihypertensive effect by lowering NE level and attenuating neural excitotoxicity in the RVLM of SIH

**Fig. 3:**
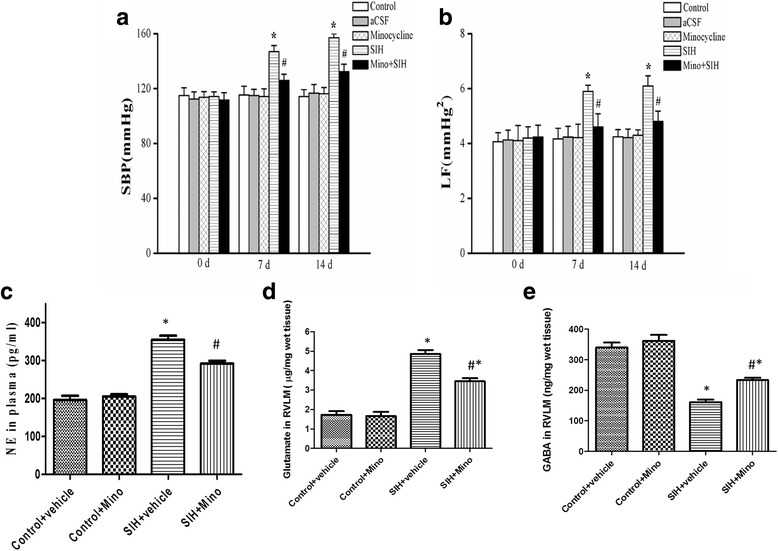
Temporal changes of systolic blood pressure (SAP; **a**) or the power density of the low frequency (LF) component of SBP spectrum (**b**) after intracisternal infusion of minocycline for 1 week in SIH. SIH rats showed higher level of NE (pg/ml) in plasma than that of control (**c**), which was attenuated by minocycline microinjection. SIH rats showed higher levels of and glutamate (**d**) and lower level of GABA (**e**) in the RVLM. One week of intracisternal infusion minocycline attenuated the decrease in RVLM GABA and the increase in glutamate in SIH rats. Values are mean ± SEM of 12 animals in each group. Statistical analysis was performed using one-way ANOVA. *P < 0.05 vs. the control group, #*P* < 0.05 vs. the SIH group, respectively. *n* = 12 (or 13) in the post hoc Scheffé multiple range analysis

Electric foot shock stressors with noise interventions in rats increased blood pressure and sympathetic vasomotor tone. From the seventh day of the stress exposure, the SBP of the SIH group kept rising gradually until stabilizing around the 14th day (from 109.50 ± 0.77 mmHg to154.83 ± 3.76 mmHg; Fig. S1). In comparison to the control group, animals receiving stressors for 2 weeks induced a significant increase (control vs. SIH, 118.00 ± 2.00 mmHg vs. 154.58 ± 3.32 mmHg, *n* = 10, *P* < 0.05) in SBP (Fig. 3a), accompanied by an increase in the sympathetic vasomotor activity reflected by the increase in power density of the LF component (control vs. SIH, 4.1 ± 1.1 mmHg^2^ vs. 5.8 ± 1.2 mmHg^2^, *n* = 10, *P* < 0.05) of the SBP spectrum (Fig. 3b). While intracisternal infusion of minocycline for 1 week attenuated stress-induced hypertension and decreased in the sympathetic vasomotor activity (Fig. 3a, b). Microinjection of minocycline or aCSF alone resulted in no apparent change in SBP and the LF component normotensive rats. Changes of body weight, mean atrial pressure (MAP), and HR at the end of the second week of the experiment were shown in Table 1. We observed that SIH rats have higher level of NE (pg/ml) in the plasma than that of control (204.8 ± 13.4 vs. 385.4 ± 29.5, *n* = 12, *P* < 0.05), which were attenuated by minocycline microinjection (SIH vs. SIH + Mino 385.4 ± 29.5 vs. 288.4 ± 19.9, *n* = 10, *P* < 0.05), suggesting that the sympathetic activity was decreased when the neuroinflammation was inhibited (Fig. 3c). SIH rats showed higher levels of glutamate (Fig. 3d) and lower level of GABA (Fig. 3e) in the RVLM, while minocycline, an inhibitor of microglia activation, attenuated the decrease in GABA and the increases in glutamate of SIH rats (Fig. 3d, e).

**Table 1:** The body weight, mean atrial pressure (MAP), and heart rate (HR) in different groups at the end of the second week of the experiment

Parameters	Control + vehicle	Control + Mino	SIH + vehicle	SIH + Mino
Body weight, g.	350 ± 8	346 ± 7	352 ± 6	345 ± 6
MAP, mmHg	109 ± 5	102 ± 6	138 ± 6^*^	125 ± 5^#^
HR, bpm	360 ± 9	354 ± 7	385 ± 6^*^	380 ± 6

Values are mean ± SEM of 12 animals in each group

*P < 0.05 vs. control rats (control + vehicle or control + Mino)

#*P* < 0.05 vs. the SIH + vehicle group in the post hoc Scheffé multiple range analysis

### Chronic stressors for 2 weeks induced increased accumulation of autophagic vacuoles in the RVLM neurons of SIH rats

**Fig. 4:**
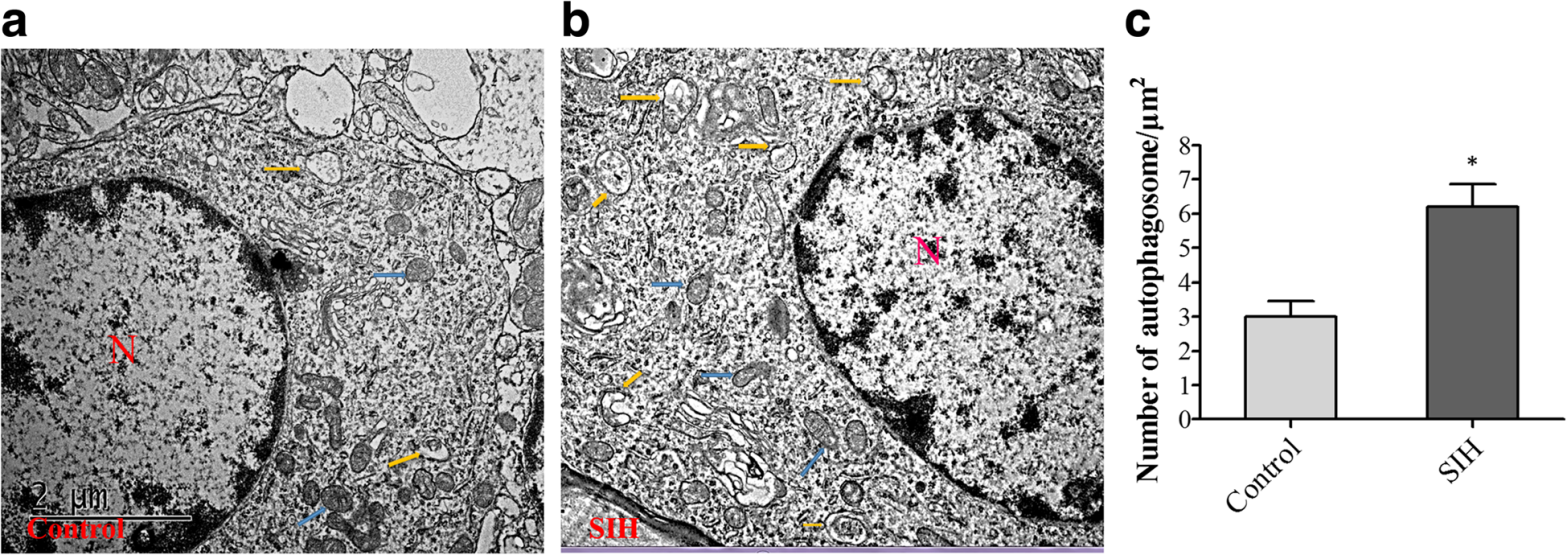
Transmission electronic microscopy photomicrographs of the RVLM neurons in the control and SIH groups. The control group showed a number of normal ribosomes, rough endoplasmic reticulum, mitochondrion, and a few autophagic bodies (**a**). The SIH group showed an increased double membrane structure containing the mitochondrion (**b**) in comparison with the control (**c**). AVs are marked with the *yellow arrow* and *blue arrows* indicate the mitochondrion. *N* nucleus. *Scale bar*, 2 μm. Values are mean ± SEM of four animals in each group. *P < 0.05 vs. control rats in the post hoc Scheffé multiple range analysis

Autophagic vacuoles (AVs) existing in the RVLM neurons in vivo was detected by transmission electron microscopy (TEM). Typical AVs contained membrane-bound vacuoles and a mitochondrion [20]. In the control group, few AVs were observed in the RVLM neurons. The normal neurons contain a number of ribosomes, rough endoplasmic reticulum, and mitochondrion (Fig. 4a). An accumulation of AVs (containing mitochondria) and few lysosomal bodies occurred in neurons in the SIH group (Fig. 4b). The number of AVs in control and SIH rats was shown in Fig. 4c.

**Fig. 5:**
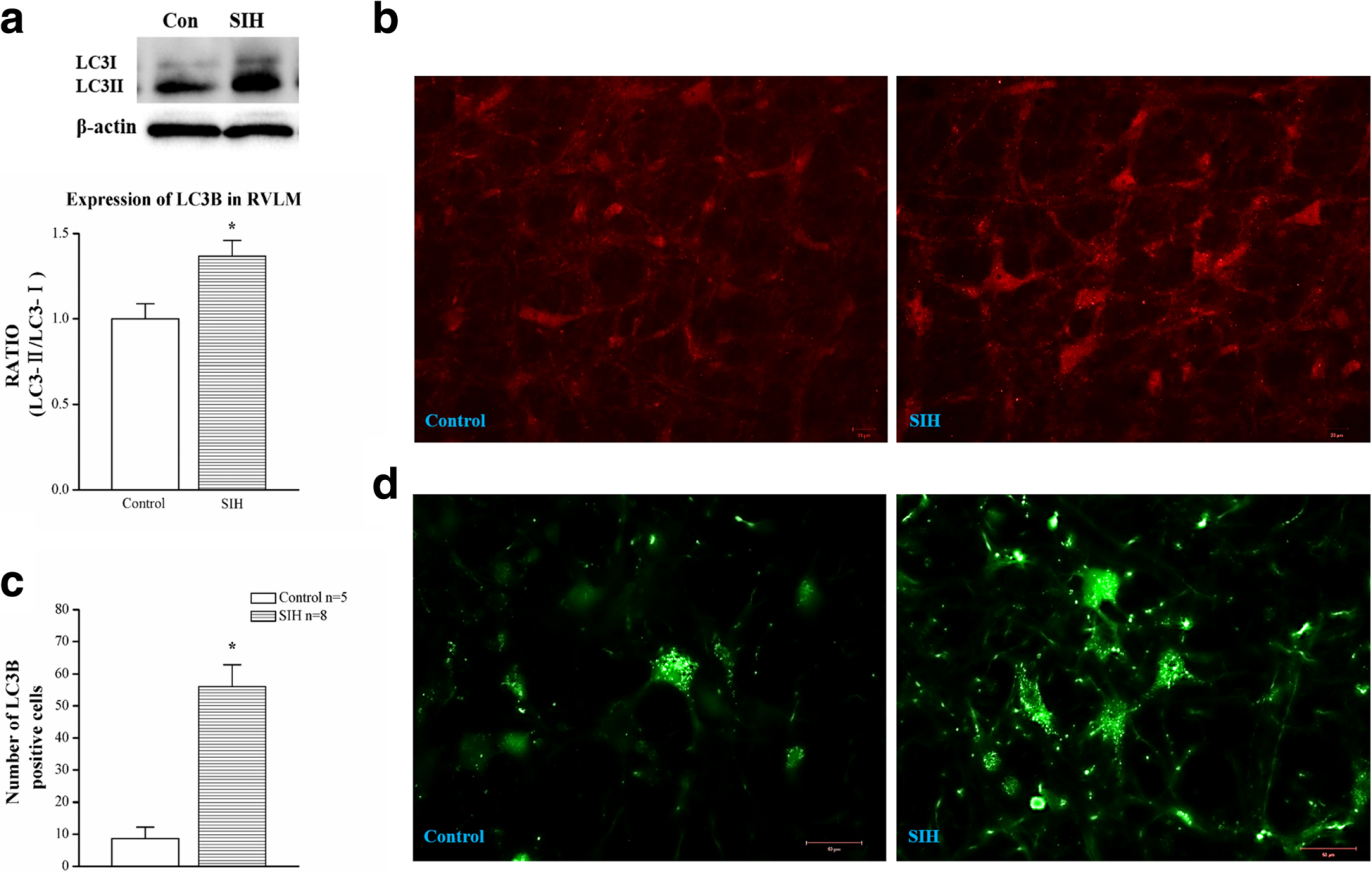
LC3 expression analysis. **a** Representative gels (*inset*) or densitometric analysis of ratio between light chain 3 (LC3)-II and LC3-I protein level in different groups. **b** Representative immunofluorescent photomicrographs for LC3B. **c** Qualitative analysis of the number of LC3B detected from RVLM neurons of SIH or normotensive rats. **d** Representative photomicrographs of LC3 dots in rats when the GFP-LC3 adeno-associated virus was transfected into the RVLM, implying the enhancement of autophagosomes in SIH. Values are mean ± SEM of quadruplicate analyses on samples pooled from five to six animals in each group. *P < 0.05 vs. control rats in the post hoc Scheffé multiple range analysis. *RVLM* rostral ventrolateral medulla. *Scale bar*, 20 μm in **b** and 40 μm in **d**

Western blot showed the autophagosome marker LC3-II/LC3-I ratio was increased (Fig. 5a). Immunofluorescent staining (Fig. 5b) also showed the number of LC3 immuno-positivities was increased (Fig. 5c) in the RVLM neurons of SIH rats. Furthermore, transfected AAV9- -GFP-LC3 dots were coincidently increased in the RVLM neurons of SIH (Fig. 5d).

### Autophagic flux blockage was observed in the RVLM, while it was facilitated by minocycline, a microglia activation inhibitor

**Fig. 6:**
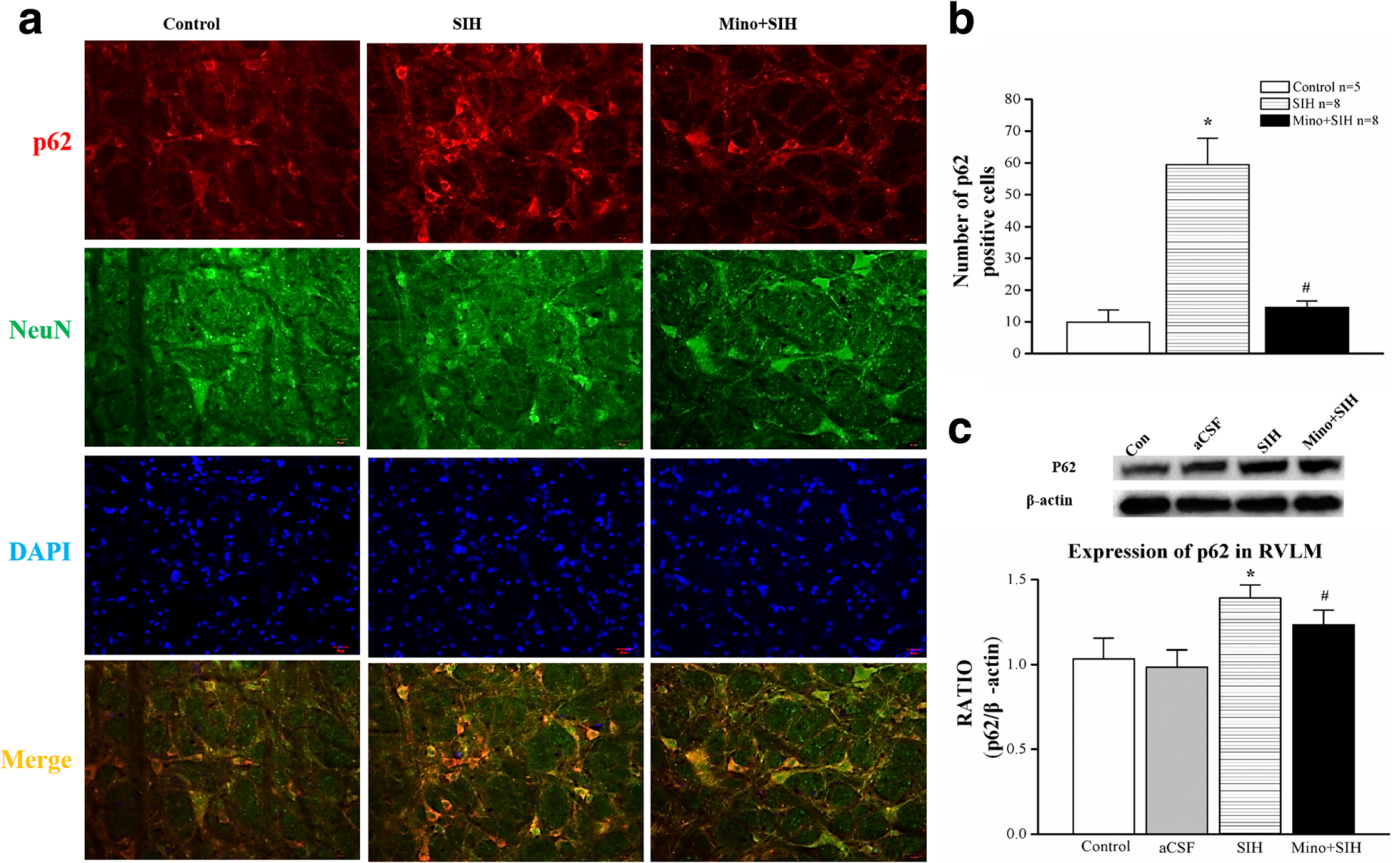
The expression of p62 in RVLM neurons. **a** Representative double immunofluorescent staining for p62 and neurons marker NeuN. **b** Qualitative analysis of the number of p62 immuno-positivities detected from RVLM neurons of SIH or normotensive rats or intracisternal infusion minocycline for 1 week in SIH. **c** Representative gels (*inset*) or densitometric analysis of p62 protein with an image analyzer. *Bar* represents mean ± SEM from five to eight rats in each group. Statistical analysis was performed using one-way ANOVA. *P < 0.05 vs. the control group, #*P* < 0.05 vs. the SIH group. *RVLM* rostral ventrolateral medulla. *Scale bar* 20 μm

**Fig. 7:**
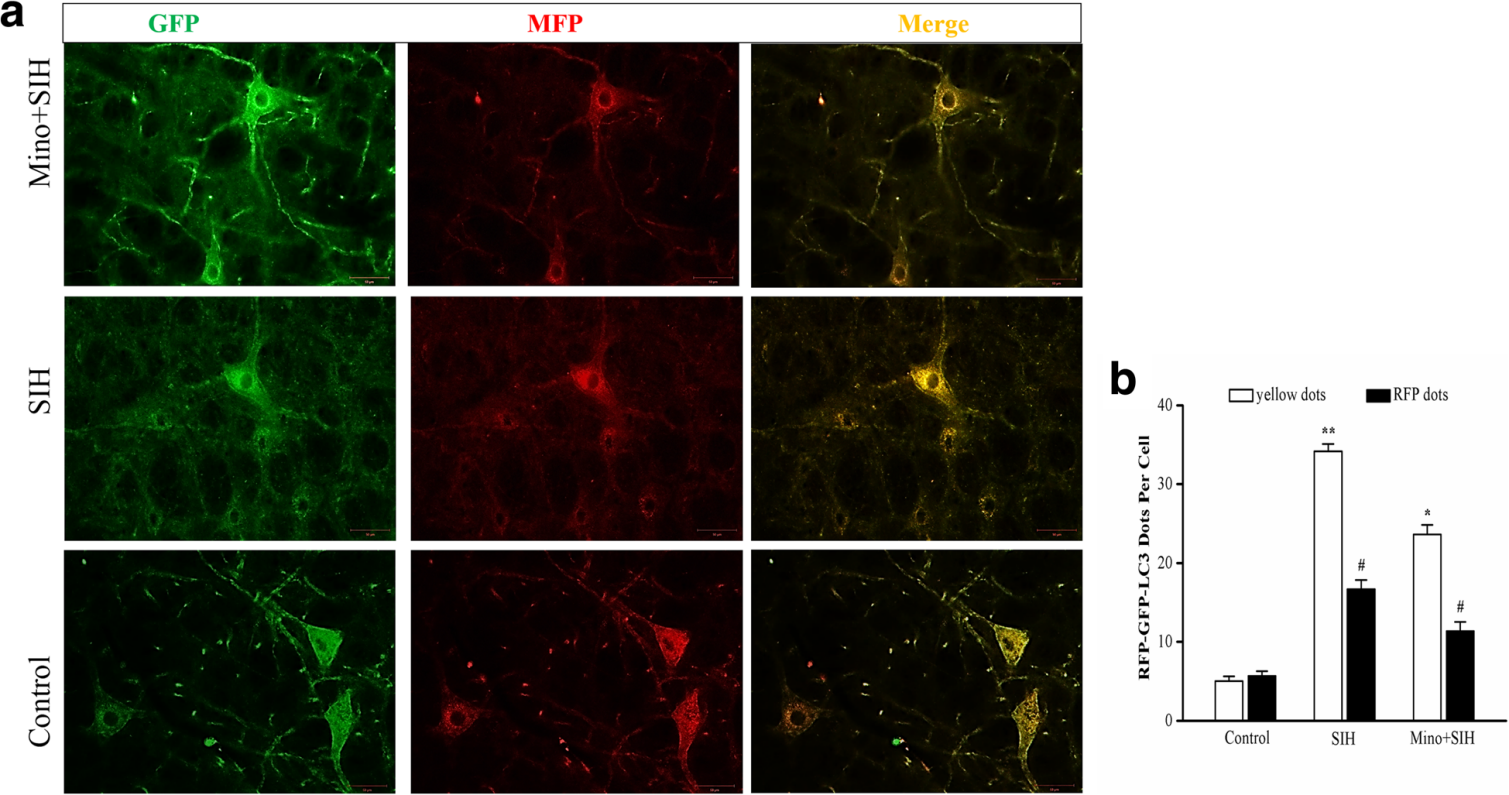
LC3 dots were visualized under fluorescent confocal microscope (**a**) and quantified (**b**) following RFP-GFP-tandem fluorescent LC3 adeno-associated virus transfected to RVLM for 2 weeks. At least 20 cells per group were included for the counting of RFP- and GFP-LC3 puncta. Statistical analysis was performed using one-way ANOVA. *P < 0.05 vs. the control group, #*P* < 0.05 vs. the SIH group. *RVLM* rostral ventrolateral medulla. *Scale bar*, 50 μm

**Fig. 8:**
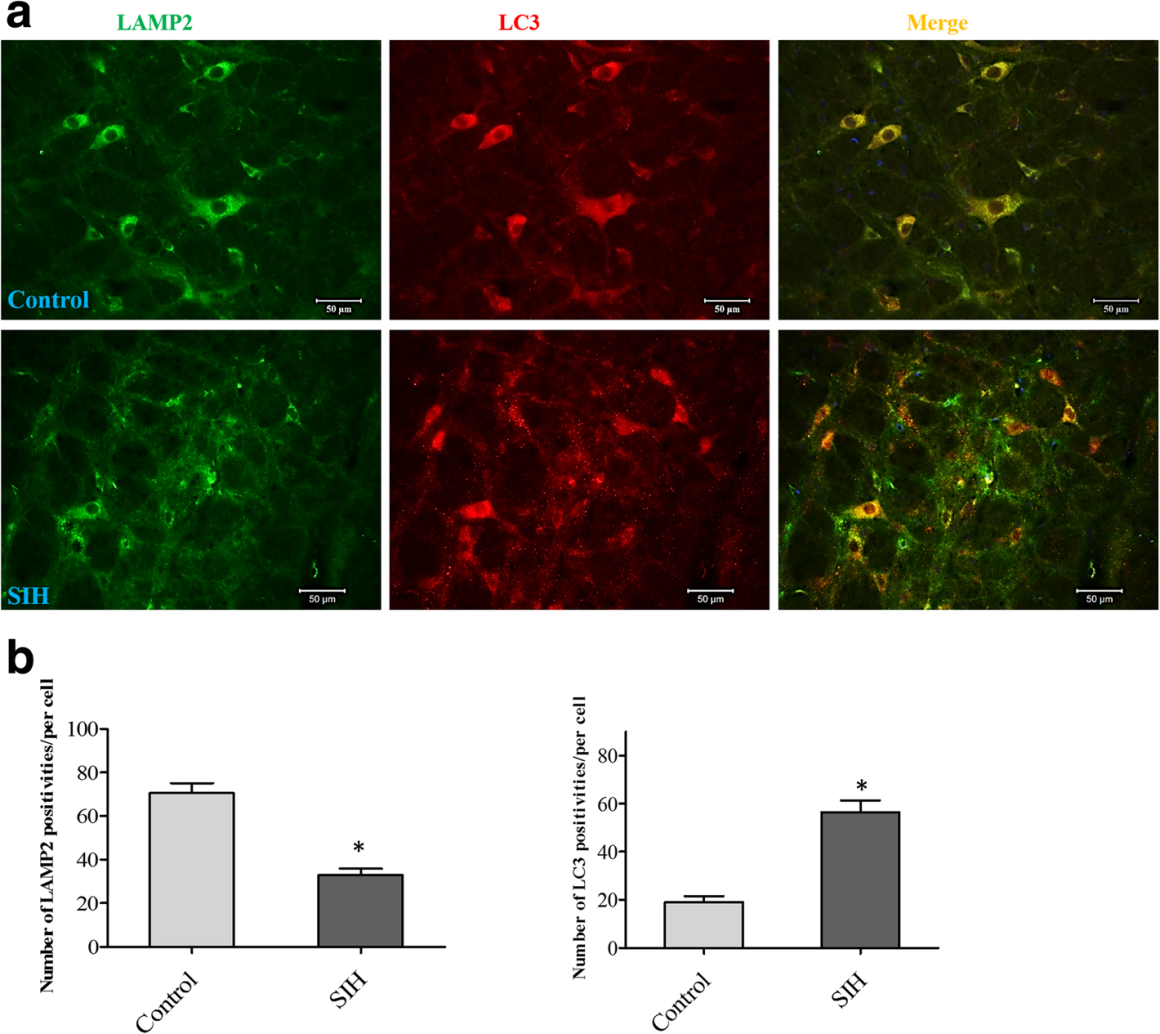
Double immunofluorescent staining showed LC3 and lysosomal marker LAMP2 was co-localized in RVLM of control and SIH rats. **a** Representative double immunofluorescent staining for LC3 and LAMP2. **b** Qualitative analysis of the number of LC3 and LAMP2 immuno-positivities detected in RVLM. *Bar* represents mean ± SEM from five to eight rats in each group. Statistical analysis was performed using one-way ANOVA. *P < 0.05 vs. the control group. *Scale bar*, 50 μm. *RVLM* rostral ventrolateral medulla, *LAMP2* lysosomal-associated membrane proteins 2

The concurrent increase of the autophagic marker LC3-II (Fig. 5) and the autophagy selective substrate p62 (Fig. 6) suggested a blockage of autophagic flux. We then monitored the autophagic flux by using the RFP-GFP-tandem fluorescent LC3 (tf-LC3) method. The basis of this method lies in the higher sensitivity of GFP fluorescence to the acidic conditions of the lysosome lumen relative to RFP. The normal RVLM neurons showed some degree of basal autophagic flux as revealed by a few yellow/red dots staining (Fig. 7a). Chronic stress exposure led to an obvious increase in the number of yellow dots per cell (Fig. 7b), indicating the blockage of autophagic flux in SIH. Furthermore, SIH rats showed a decreased expression of lysosomal-associated membrane proteins 2 (LAMP2), which might be one of the causes for the autophagic flux blockage (Fig. 8). Intracisternal infusion of minocycline significantly decreased the yellow dots in the RVLM neurons (Fig. 7a, b) in SIH, which indicated that inhibition of microglia activation in the RVLM facilitated autophagic flux in SIH rats.

## Discussion

Activation of sympathetic neural pathways is an important mediator of acute and chronic stress-induced hypertension and heart disease, and increased sympathetic activity resulting from repetitive psychogenic stress, obesity, or high sodium intake [35–37]. Stress is often defined as a state of mental, physical, or emotional tension in response to various unexpected demanding factors and/or circumstances [5]. Our previous studies confirmed that stressor of electric foot shocks combining with noise resulted in elevated BP associated with stress-induced hypertension (SIH) [2–4].

An increase in sympathetic outflow to the peripheral vasculature from the rostral ventrolateral medulla (RVLM), where premotor neurons for the maintenance of sympathetic vasomotor activity are located [38], contributes to the neural mechanism of hypertension [7]. Increased sympathetic tone was associated with neuroinflammation in the RVLM. Shi et al. [12] confirmed that microglia were the major players responsible for the development of neuroinflammation and modulating neuronal excitation. Microglial activation and production of PICs activated the renin-angiotensin system within the hypothalamus, which play a role in elevated blood pressure [39]. We observed the increased levels of activated microglia and PICs in the RVLM in animal models of SIH, which was consistent with the similar previous study [12, 39]^.^ It was reported that the interactions between activated micro- and astroglia result in a significant production of TNF-α, followed by a release of glutamate [40]. All these factors are capable of initiating propagating waves of Ca^2+^ excitation within astroglial networks and modulating the excitability of local neuronal circuits [41–43].

Our results demonstrated that stress induced the accumulation of autophagic vescoles (AVs) containing mitochondria in the RVLM neurons. As we know, mitochondria fission segregates damaged mitochondria from intact ones, where the damaged part of mitochondria is subjected to mitophagy whereas the intact part to fusion [44]. Evidence has implicated autophagic dysfunction in the pathogenesis of several major neurodegenerative disorders, such as Parkinson’s disease and Alzheimer’s disease, where deficient elimination of abnormal and toxic protein aggregates promotes cellular stress, failure, and death. In addition, autophagy has also been found to affect metal-induced neurotoxicity [45].

The ability to monitor autophagic flux in tissues and tumors in vivo remains difficult, and at present, the surrogates most commonly used includes immunohistochemistry for LC3B and the autophagy substrate p62/Sqstm1. However, part of LC3 II is destroyed together with cargo in autophagolysosomes, so its levels can be both increased or decreased in the conditions of upregulated autophagy. P62 was degraded in autolysosomes, thus serving as readout of autophagic degradation [22, 23]. Punctate LC3 staining and lower p62 staining suggested ongoing autophagic flux; in contrast, the concurrent increase of LC3-II and p62 suggested a blockage of autophagic flux.

In the present study, we observed that stress diffuse LC3 staining and increased p62 which suggested a reduced or impaired autophagic flux. We also monitored the autophagic flux by using the RFP-GFP-tandem fluorescent LC3 (tf-LC3) method, which the RVLM was transfected with a tf-LC3 adeno-associated virus. The basis of this method lies in the higher sensitivity of GFP fluorescence to the acidic conditions of the lysosome lumen relative to RFP. The normal RVLM neurons showed some degree of basal autophagic flux, while chronic stress exposure led to the blockage of autophagic flux in SIH probably due to either defective autophagosome fusion with lysosome or compromised lysosomal function. So it was not surprising that we found SIH rats showed a decreased expression of lysosomal-associated membrane proteins 2, which might be one of the causes for the autophagic flux blockage.

We propose that RVLM microglia cells are activated and release PICs, which resulted in autophagic flux blockage thereby directly or indirectly increasing the activity of RVLM sympathetic neurons. The available evidence is: firstly, the expression of activated microglia and PICs in the RVLM is increased in the RVLM of SIH animal models; secondly, we provide evidence that the autophagic flux is blocked with decreased lysosomal-associated membrane protein 2 in SIH rats. Thirdly, intracisternal infusion of minocycline decreases blood pressure, sympathetic outflow, and increase autophagic flux in the RVLM neurons of SIH rats. Minocycline, a second-generation tetracycline, readily crosses the blood-brain barrier [28]; we did not test higher doses as minocycline crosses the blood-brain barrier, and increasing doses increase the likelihood of peripheral actions of minocycline. In addition to anti-inflammation, minocycline and other tetracycline derivatives are proposed to attenuate apoptosis and inhibit production of reactive oxygen species via an action on mitochondria [46].

Our results showed that SIH rats had higher levels of excitatory amino acid glutamate and lower level of inhibitory amino acid GABA in the RVLM. The disturbance of normal autophagic flux might, thus, result in the accumulation of excitatory amino acids in the RVLM to induce sympathoexcitation. Minocycline attenuated the decrease in GABA and the increases in glutamate in the RVLM of SIH rats. It was reported that minocycline had significant effects on dopamine and glutamate transmission [47]; the detail mechanisms of minocycline inducing amino acid releasing need to be further clarified. We supposed that stress might induce autophagic flux blockage thereby increase oxidative stress and modulating the neural excitotoxicity. Our present observations complement previous findings [12, 39]. During the processes of autophagy, autophagosome-lysosome fusion is important for the degradation of autophagosome content; several groups of proteins involved in membrane fusion also play a role in late-stage autophagy [48]. Further understanding of the regulation of autophagy by cytokines and chemokines signaling may lead to recognition of therapeutic targets for SIH. Thus, microglia-related autophagy in the RVLM cardiovascular control centers may have a key role in the development of neurogenic hypertension. To pinpoint the pathways and molecules which mediate the microglia-neuron interaction may provide potential targets for the management of SIH.

## Conclusions

Collectively, the present study suggested that neuroinflammation might induce autophagic flux blockage and cause neural excitotoxicity in the RVLM neurons of rats following SIH. Furthermore, minocycline, an anti-inflammatory agent and inhibitor of microglial activation, alleviated autophagic flux blockage and neural excitotoxicity, which elicited an antihypertensive effect in the SIH rat model.

**Fig. 9:**
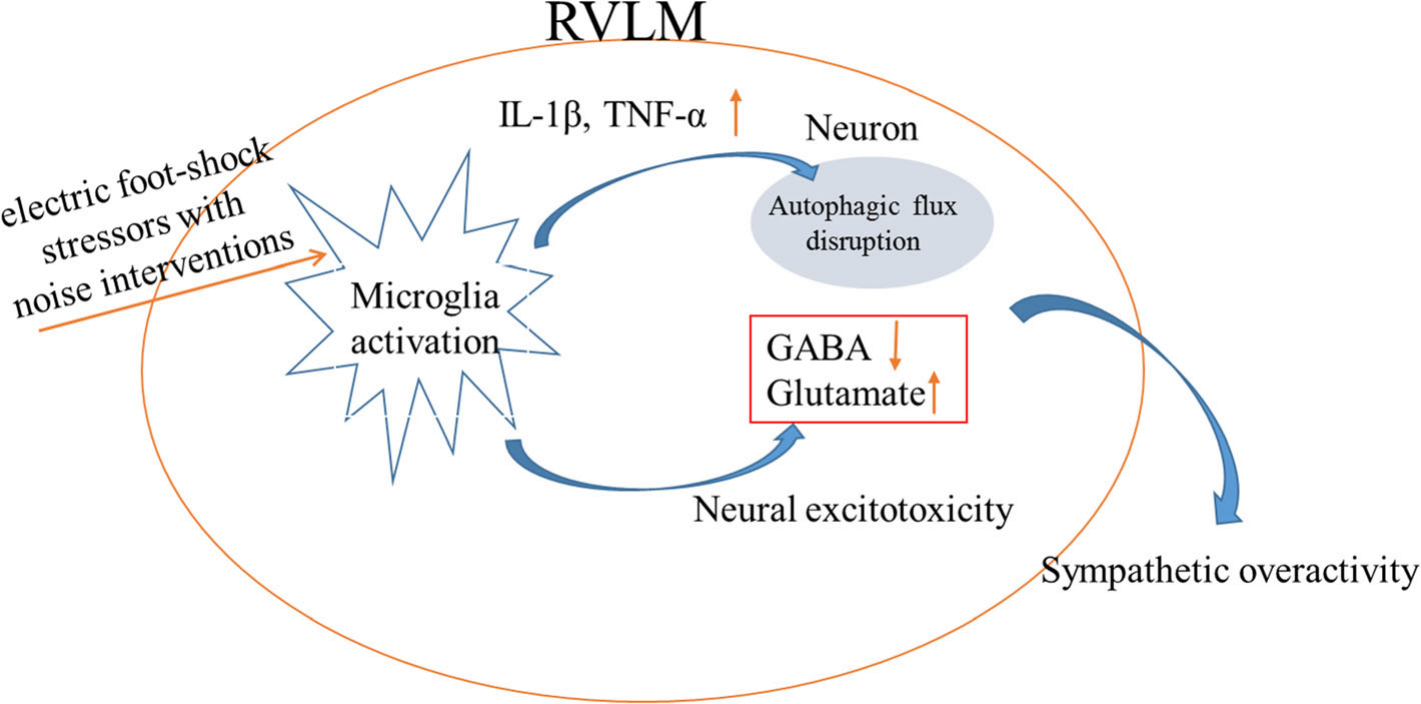
Schematic diagrams illustrating the putative mechanisms that enhanced microglial PICs contributed to autophagic flux disruption and caused the neural excitotoxicity in the RVLM neurons

The schematic diagram illustrating the putative mechanisms that enhanced microglial PICs contributed to autophagic flux disruption and caused the neural excitotoxicity in the RVLM neurons (Fig. 9).

### Limitations

Firstly, the relationship between neuroinflammation and neuron autophagic flux was focused currently. Autophagy modulator, e.g., rapamycin bafilomycin A1, chloroquin, or 3-methyladenine (3-MA), on blood pressure was not investigated. Much more experiments in vivo and in vitro will be conducted in future series studies even though we found rapamycin, an autophagy inducer, attenuated the decrease in GABA and the increase in glutamate of SIH rats (Additional file 4: Figure S4). Secondly, we used intracisternal infusion minocycline instead of RVLM microinjection directly because minocycline readily crosses the blood-brain barrier. Increasing doses increase the likelihood of peripheral actions of minocycline, so low dose and chronic administration is requested. It is very hard to chronically implant and fix the minipump in the RVLM. Directly interfering with the neuroinflammtion in the RVLM to observe its effect on autophagy might be essential.

## Additional file

Additional file 1: Figure S1. Effects of chronic stress application on temporal changes of SBP. Data demonstrated that stressed rats underwent a significant increase in tail artery systolic blood pressure. Values are mean ± SEM; Statistical analysis was performed using one-way ANOVA. **P* < 0.01, ***P* < 0.05, vs control group, #*P* < 0.05, vs SIH group, respectively. *n* = 12 (or 13). SBP, systolic blood pressure. (TIFF 170 kb)
Additional file 2: Figure S2. Photomicrographs of non-activated and activated microglia taken from the RVLM of SIH. The photo of activated microglia (red arrow, B) was taken from within the RVLM and the photomicrograph of the non-activated microglia (yellow arrow, A) was taken from the area adjacent to the RVLM in the same rat. Magnification was 200× for both shots. Scale bar = 50 μm. (TIFF 2495 kb)
Additional file 3: Figure S3. Identification of the RVLM microinjection sites. (A and B) represented the photomicrograph and schematic graphs, respectively. The arrow in (A) and the black arrow in (B) indicated the microinjection sites of RVLM. (TIFF 1370 kb)
Additional file 4: Figure S4. showed the effect of rapamycin, an autophagy inducer, on amino acid releasing in different group rats. Rapamycin attenuated the decreasing in GABA and the increasing in glutamate of SIH rats. Values are mean ± SEM; Statistical analysis was performed using one-way ANOVA. **P* < 0.05, vs control group, #*P* < 0.05, vs SIH group, respectively. *n* = 10. (TIFF 129 kb)

## Abbreviations

aCSF: Artificial cerebrospinal fluid
AVs: Autophagic vacuoles
BP: Blood pressure
CNS: Central nervous system
GABA: Gamma aminobutyric acid
HPLC: High-performance liquid chromatography
HR: Heart rate
Iba1: Ionized calcium-binding adaptor molecule-1
LAMP2: Lysosomal-associated membrane proteins 2
LC3: Microtubule-associated protein 1 light chain 3
LF: Power density of the low frequency component of the SBP spectrum
MAP: Mean atrial pressure
Mino: Minocycline
mRFP-EGFP-LC3: Monomeric red fluorescence protein-enhanced green fluorescence protein-LC3
NE: Norepinephrine
p62: Poly-ubiquitin binding protein sequestosome 1
PICs: Pro-inflammatory cytokines
RVLM: Rostral ventrolateral medulla
SBP: Systolic blood pressure
SIH: Stress-induced hypertension
SNS: Sympathetic nervous system
TEM: transmission electron microscopy
tf-LC3: RFP-GFP-tandem fluorescent LC3
