# Supplementary figures and images for: Neuroinflammation contributes to autophagy flux blockage in the neurons of rostral ventrolateral medulla in stress-induced hypertension rats
Source: J Neuroinflammation. 2017 Aug 23;14:169. doi: 10.1186/s12974-017-0942-2 (PMC5569471; doi:10.1186/s12974-017-0942-2)

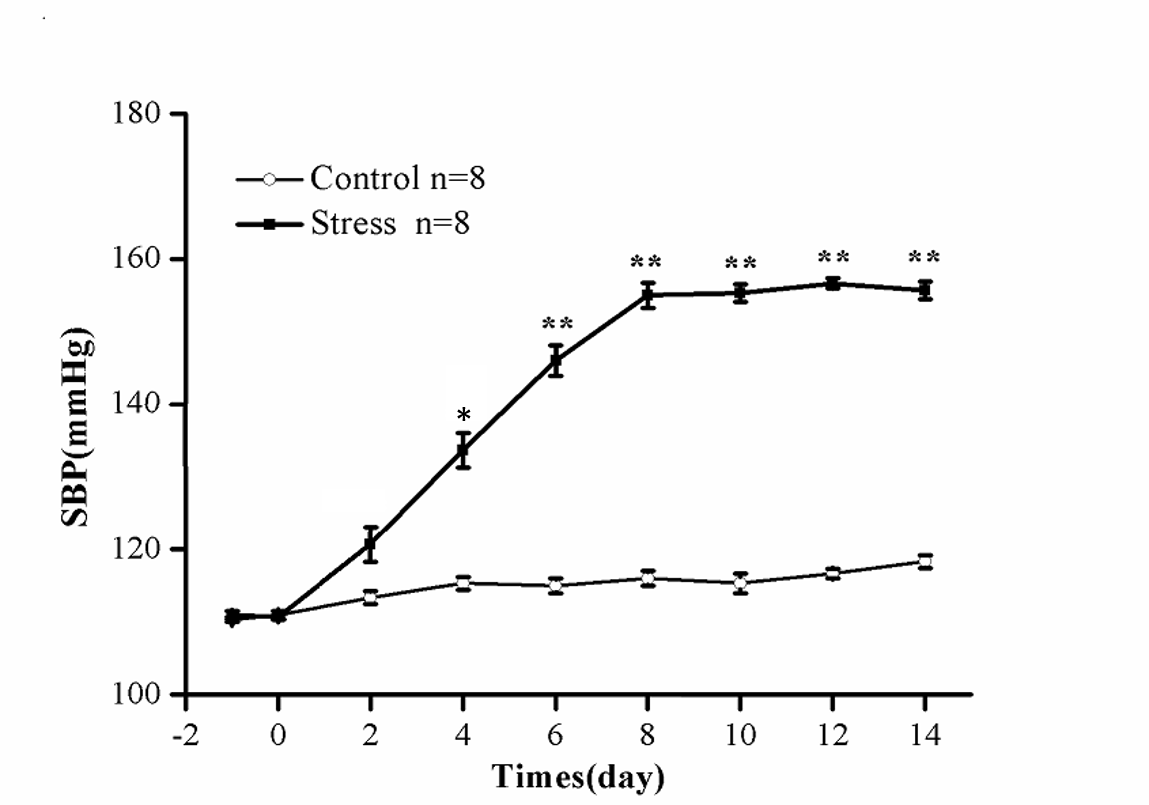

Supplement: Additional file 1: Figure S1. — Effects of chronic stress application on temporal changes of SBP. Data demonstrated that stressed rats underwent a significant increase in tail artery systolic blood pressure. Values are mean ± SEM; Statistical analysis was performed using one-way ANOVA. *P < 0.01, **P < 0.05, vs control group, #P < 0.05, vs SIH group, respectively. n = 12 (or 13). SBP, systolic blood pressure. (TIFF 170 kb) [file 12974_2017_942_MOESM1_ESM.tif]

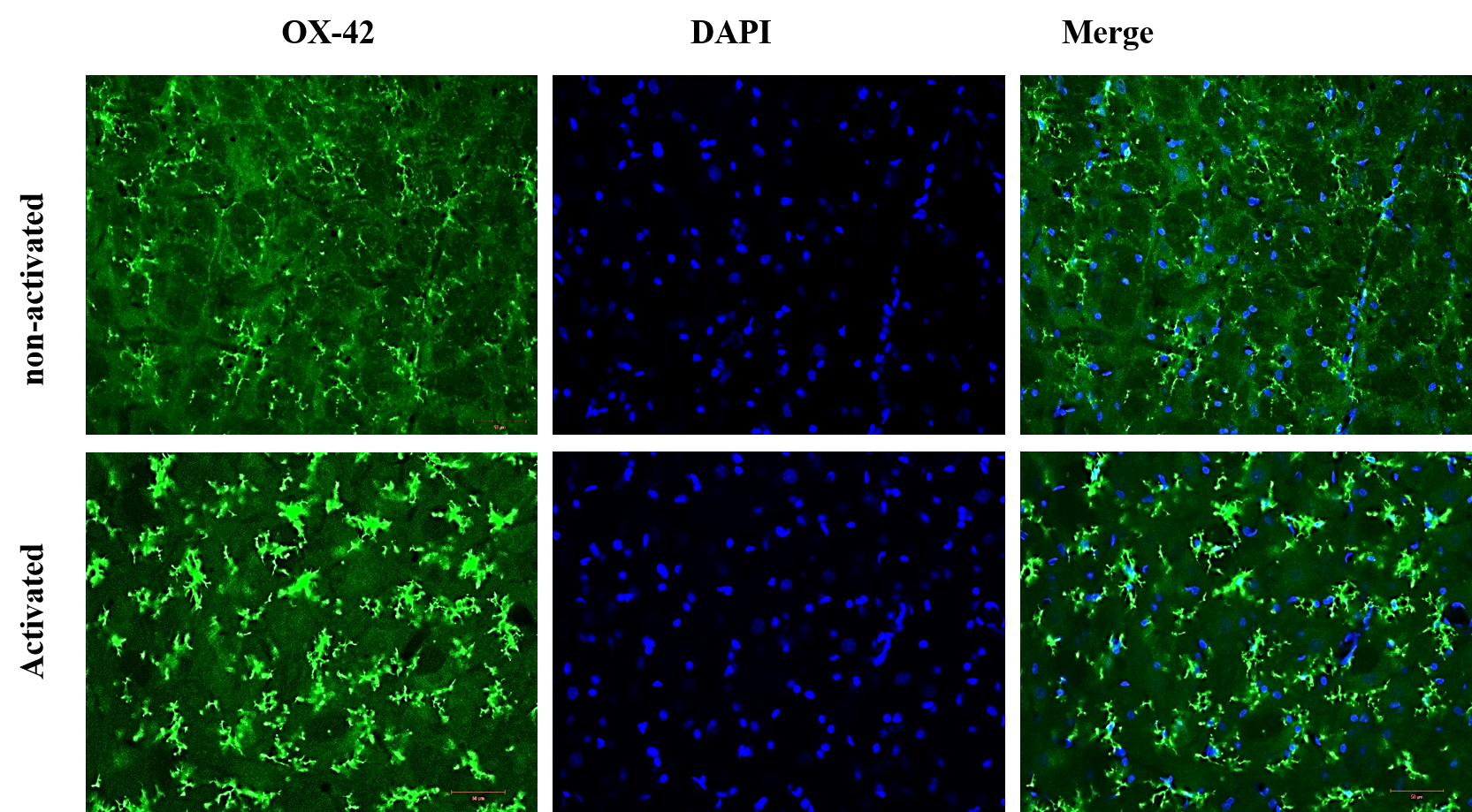

Supplement: Additional file 2: Figure S2. — Photomicrographs of non-activated and activated microglia taken from the RVLM of SIH. The photo of activated microglia (red arrow, B) was taken from within the RVLM and the photomicrograph of the non-activated microglia (yellow arrow, A) was taken from the area adjacent to the RVLM in the same rat. Magnification was 200× for both shots. Scale bar = 50 μm. (TIFF 2495 kb) [file 12974_2017_942_MOESM2_ESM.tif]

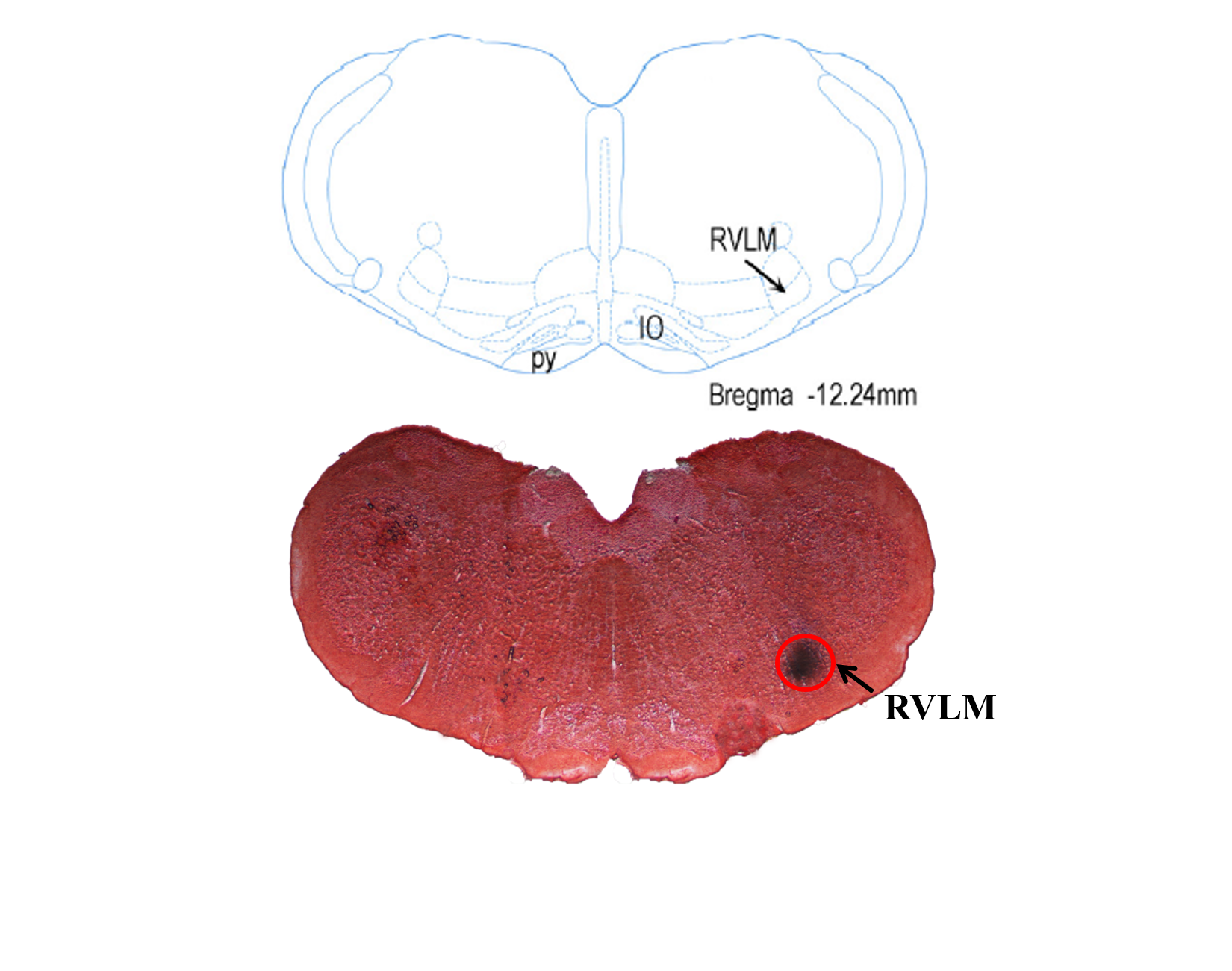

Supplement: Additional file 3: Figure S3. — Identification of the RVLM microinjection sites. (A and B) represented the photomicrograph and schematic graphs, respectively. The arrow in (A) and the black arrow in (B) indicated the microinjection sites of RVLM. (TIFF 1370 kb) [file 12974_2017_942_MOESM3_ESM.tif]

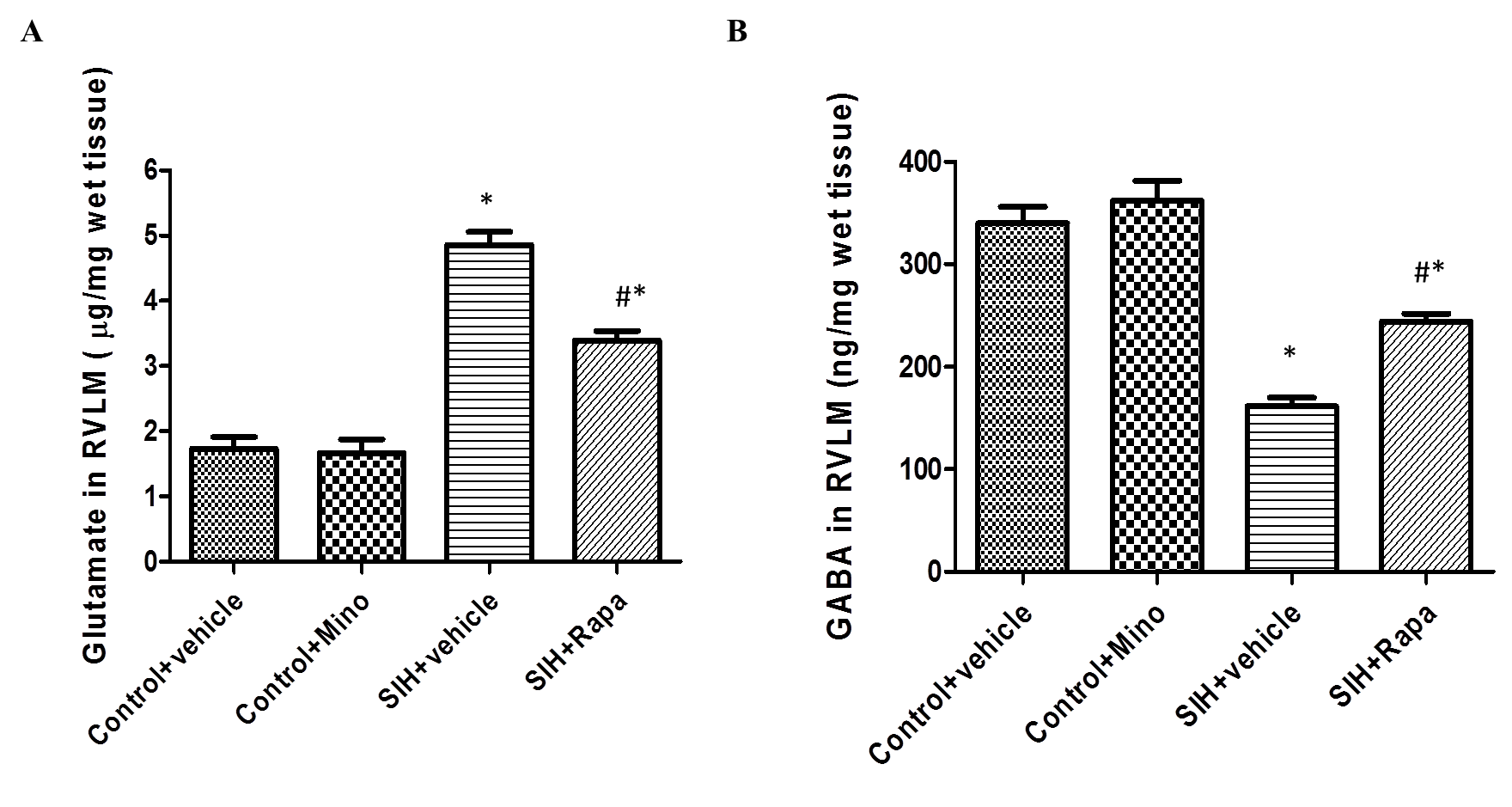

Supplement: Additional file 4: Figure S4. — showed the effect of rapamycin, an autophagy inducer, on amino acid releasing in different group rats. Rapamycin attenuated the decreasing in GABA and the increasing in glutamate of SIH rats. Values are mean ± SEM; Statistical analysis was performed using one-way ANOVA. *P < 0.05, vs control group, #P < 0.05, vs SIH group, respectively. n = 10. (TIFF 129 kb) [file 12974_2017_942_MOESM4_ESM.tif]
